# Genetic characterization of feline panleukopenia virus from dogs in Vietnam reveals a unique Thr101 mutation in VP2

**DOI:** 10.7717/peerj.9752

**Published:** 2020-10-12

**Authors:** Minh Hoang, Cheng-Nan Wu, Chuen-Fu Lin, Huong Thanh Thi Nguyen, Van Phan Le, Ming-Tang Chiou, Chao-Nan Lin

**Affiliations:** 1 Department of Veterinary Medicine, College of Veterinary Medicine, National Pingtung University of Science and Technology, Pingtung, Taiwan; 2 Department of Anatomy and Histology, College of Veterinary Medicine, Vietnam National University of Agriculture, Hanoi, Vietnam; 3 Department of Medical Laboratory Science and Biotechnology, Central Taiwan University of Science and Technology, Taichung, Taiwan; 4 Department of Veterinary Medicine, College of Veterinary Medicine, National Chiayi University, Chiayi, Taiwan; 5 Department of Microbiology and Infectious Disease, College of Veterinary Medicine, Vietnam National University of Agriculture, Hanoi, Vietnam; 6 Animal Disease Diagnostic Center, College of Veterinary Medicine, National Pingtung University of Science and Technology, Pingtung, Taiwan

**Keywords:** Feline parvovirus, Canine parvovirus type 2, Sequences analysis, 3D structure

## Abstract

**Background:**

Canine parvovirus type 2 (CPV-2) and feline parvovirus (FPV) are known as the main causes of several serious diseases and have a severe impact on puppies and kittens, respectively. FPV and new CPV-2 variants are all able to infect cats, causing diseases indistinguishable from feline panleukopenia. However, FPV only replicates efficiently in feline cells in vitro and replicates in dogs in the thymus and bone marrow without being shed in feces. In our previous study, the genotypes of six parvoviral isolates were unable to be identified using a SimpleProbe^®^ real-time PCR assay.

**Methods:**

In the present study, we characterized previously unidentified FPV-like viruses isolated from dogs in Vietnam. The six isolates were utilized to complete VP2 gene sequencing and to conduct phylogenetic analyses.

**Results:**

Sequence analysis of the six parvoviral strains identified the species as being similar to FPV. Phylogenetic analysis demonstrated that the complete VP2 genes of the strains are similar to those of FPV. The FPV-like strains contain a Thr101 mutation in the VP2 protein, which is different from prototype FPV strains.

**Discussion:**

Our data provide evidence for the existence of changes in the charge, protein contact potential and molecular surface of the core of the receptor-binding size with an Ile101 to Thr101 mutation. This is also the first study to provide reliable evidence that FPV may be a threat to the Vietnamese dog population.

## Introduction

Canine parvoviral enteritis is characterized by acute gastroenteritis and/or bloody diarrhea and is one of the most common infectious diseases in puppies (Appel, Scott & Carmichael, 1979; Black et al., 1979). The causative agent canine parvovirus type 2 (CPV-2) is a single-stranded, nonenveloped, small DNA virus with a genome size of approximately 5 kb (Reed, Jones & Miller, 1988). It is antigenically and genetically unrelated to canine parvovirus type 1, which is currently called canine minute virus (Appel, Scott & Carmichael, 1979). CPV belongs to the genus *Protoparvovirus* in the family *Parvoviridae* and according to the International Committee on Taxonomy of Viruses, it is included with the species Carnivore *protoparvovirus* 1 together with feline parvovirus virus (FPV), mink enteritis virus (MEV) and raccoon parvovirus (RPV) (Cotmore et al., 2019).

FPV was first identified to have a viral cause in 1928 (Verge & Cristoforoni, 1928) and isolated in tissue culture in 1964 (Johnson, 1965). CPV-2 is believed to have originated from FPV or a closely related FPV-like parvovirus of wild carnivores (Allison et al., 2014); however, these viruses are very different in terms of host cell specificity. CPV-2 can replicate in both canine and feline cells in vitro and in vivo (Truyen & Parrish, 1992). Conversely, FPV can only replicate efficiently in the thymus but loses its efficiency in mesenteric lymph nodes or in the small intestine of FPV-inoculated dogs (Truyen & Parrish, 1992). CPV-2 shows several missense mutations within the VP2 protein compared to FPV, including Lys80Arg, Lys93Asn, Val103Ala, Val232Ile, Asp323Asn, Asp375Asn, Asn564Ser and Ala568Gly (Parrish, 1991; Parrish, Aquadro & Carmichael, 1988; Truyen et al., 1995). Shortly after CPV-2 was first identified in the late 1970s, CPV-2 was replaced in the dog population by strains carrying small antigenic variations of the VP2 protein (Decaro & Buonavoglia, 2012). Variants CPV-2a, 2b and 2c can be distinguished by monoclonal antibodies and molecular analysis based on residue 426 of the VP2 protein (Decaro et al., 2005; Parrish et al., 1991; Parrish et al., 1985). The functions of VP2 protein include facilitating transferrin receptor binding (Chang, Sgro & Parrish, 1992), controlling host range (Chang, Sgro & Parrish, 1992) and eliciting an immune response (Lopez de Turiso et al., 1991). Transferrin receptor type-1 (TfR) serves as a receptor for FPV and CPV-2 infection (Hueffer et al., 2003a). Physiologically, TfR mediates iron uptake into cells by binding and importing iron-loaded transferrin, which also binds to the hereditary hemochromatosis protein in the intestine to regulate iron uptake by blocking transferrin binding (Kawabata, 2019). Apical domain residues are reported to be critical for controlling parvovirus binding in FPV and CPV-2 interaction with canine and feline TfR, respectively (Goodman et al., 2010; Hueffer et al., 2003a; Kaelber et al., 2012; Palermo, Hueffer & Parrish, 2003).

According to research surveillance of CPV-2, a novel CPV-2c variant was identified in several Asian countries in the past few years, including China (Geng et al., 2015; Wang et al., 2016; Zhang et al., 2010; Zhao et al., 2015). , Taiwan (Chiang et al., 2016; Lin et al., 2017), Laos (Vannamahaxay et al., 2017), Thailand (Charoenkul et al., 2019) and Vietnam (Hoang et al., 2019a; Nakamura et al., 2004). An antigenic variant of this CPV-2c with a few residue substitutions (Ala5Gly, Phe267Tyr, Tyr324Ile and Gln370Arg) compared to the prototype CPV-2c was detected (Chiang et al., 2016; Geng et al., 2015; Lin et al., 2017; Wang et al., 2016; Zhao et al., 2015). This novel CPV-2c variant is prevalent on the Asian continent. Surprisingly, a few FPV strains have been detected from naturally infected dogs in Pakistan (Ahmed et al., 2018) or Thailand (Charoenkul et al., 2019). In a previous study, we developed a reliable and sensitive tool for differentiating between the CPV-2 genotypes (Hoang et al., 2019b). Interestingly, there were some parvoviral isolates with genotypes that could not be identified using this SimpleProbe^®^ real-time PCR assay (Hoang et al., 2019a), raising the question of whether any FPV variants can occur and become emerging viruses in dog populations. In the present study, we characterized previously unidentified FPV-like viruses in dogs in Vietnam. Here, the complete VP2 gene sequences and 3D structures of FPV-like viruses are analyzed and discussed.

## Materials & Methods

### Specimen preparation

Six parvoviral isolates with genotypes that could not be identified using the SimpleProbe^®^ real-time PCR assay (Hoang et al., 2019b). Details with respect to the clinical histories of individual animals are provided in Table 1.

**Table 1:** Clinical status of the 6 dogs and corresponding FPV analysis.

Strain	Sampling time	Region of Vietnam	Age^a^	Sex	Vaccination	Clinical signs	Results of the rapid test^c^	Outcome	CPV-like or FPV-like	Accession number
HN39AA	25.11.2017	North	5 M	M	No	Diarrhea, Vomiting	+	Alive	FPV-like	MK357738
HN3X	16.12.2017	North	3 M	N/A^b^	No	Diarrhea, Vomiting	+	Alive	FPV-like	MK357739
HN40AA	23.12.2017	North	4 M	M	N/A^b^	Diarrhea, Vomiting	+	Alive	FPV-like	MK357740
HN10X	13.1.2018	North	2 M	N/A	N/A	Diarrhea, Vomiting	+	Alive	FPV-like	MK357741
HN41AA	4.1.2018	North	9 M	M	N/A	Diarrhea, Vomiting	+	Alive	FPV-like	MK357742
HN11X	6.1.2018	North	3 M	N/A	N/A	Diarrhea, Vomiting	+	Alive	FPV-like	MK357743

**Notes.**

a Age of the dog at presentation (in months).

b N/A: not available.

c Canine parvovirus Ag test, GREENAGE CO., LTD, VIETMAN.

### Complete VP2 gene amplification and sequencing

The entire VP2 gene of parvovirus was PCR amplified by as described by Hoang et al. (Hoang et al., 2019a).

### Sequence and phylogenetic analysis

These six VP2 DNA sequences were compared to reference FPVs (EU659114, EU659113, AB000066, M38246, KT240134, EU659112, X55115, M24002, JX475256, HQ184195, EU498716, EU498686, EU498692, DQ474237, AB054226), CPV-2 (M38245), CPV-2a (M24000), new CPV-2a (AY742953), CPV-2b (M74852), new CPV-2b (AY742955) and CPV-2c (FJ222821, MK357736, AB120727). The Clustal W and MegAlign programs (DNASTAR, Madison, WI, USA) were utilized for multiple alignments of nucleic acid and amino acid sequence. A total of 507 complete VP2 gene sequences (FPV-like, CPV-2-like, MEV-like and RPV strains) were obtained from the National Center for Biotechnology Information database for phylogenetic analysis. Phylogenetic analyses were processed properly by the maximum likelihood methods using Molecular Evolutionary Genetics Analysis X software based on the Tamura-Nei model under the assumption of a uniform rate of evolution (Tamura et al., 2013). The initial tree of the heuristic search were obtained automatically by applying Neighbor-Join and BioNJ algorithms to a pairwise distance matrix estimated using the maximum composite likelihood (MCL) method.

### Sequence conservation analysis

Sequences conservation an amino acid levels was determined by calculating the information content of each position in a multiple alignment of 600 Carnivore protoparvoviruses, including 471 CPV-2 strains from dogs, 19 CPV-2 strains from cats, 90 FPV strains from cats and 21 FPV strains from dogs. The sequence logo of the critical VP2 amino acid residues was generated using SeqLogo in TBtools (https://github.com/CJ-Chen/TBtools).

### Analysis of the transferrin receptor-binding site of FPV capsid VP2

An FPV (MK357738) VP2 structure comprising the sequence from amino acid position 37-583 was created using homology modeling (https://swissmodel.expasy.org/) based on feline panleukopenia virus strain 193 (X55115). The 3D structure was visualized using the open-source software PyMol (https://github.com/schrodinger/pymol-open-source). Intramolecular hydrogen bonds were displayed using the action popup (via Find -¿Polar Contacts). The electrostatic distribution on the surface was generated by automated PyMol representation (via the action popup -¿Generate -¿Vacuum Electrostatics -¿Protein Contact Potential) to visualize protein contact potential.

## Results

### PCR amplification and genotype analysis

**Figure 1:**
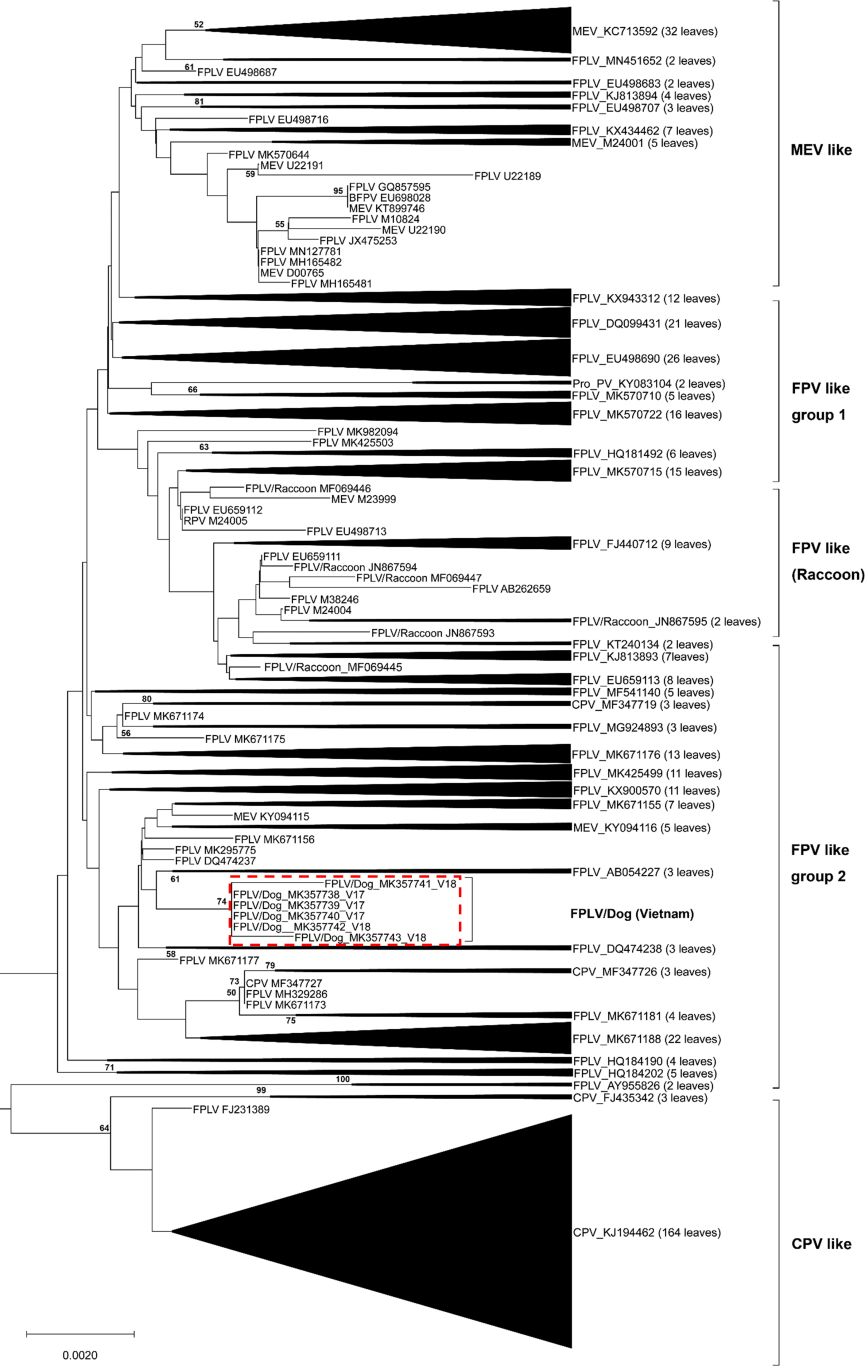
Phylogenetic relationship based on broad nucleotide sequences (*n* = 507) of the complete VP2 genes of FPV, CPV-2, MEV and RPV. Phylogenetic relationship based on broad nucleotide sequences (*n* = 507) of the complete VP2 genes of FPV, CPV-2, MEV and RPV. The phylogenetic tree was constructed using the maximum likelihood method with bootstrap analysis (*n* = 1, 000) to determine the best fitting tree. Bootstrap support values greater than 50 are shown. FPV-like isolates in the present study are indicated as a red dash line box.

The complete VP2 genes of the 6 Vietnamese untypeable parvoviral DNAs from North Vietnam were collected (Table 1). A broader phylogenetic analysis based on the nucleotide sequences of the 507 full-length VP2 gene revealed that our six parvoviral DNA sequences cluster with FPV-like groups and not CPV-2-like, MEV-like or RPV groups (Fig. 1). Interestingly, several Chinese CPV-2-like strains isolated from cats were also clustered in this group (MF347726 and MF347727). These samples were ruled out the possibility of contamination by checking the feline housekeeping gene (data not shown). Notably, the results of this study revealed the appearance of FPV-like strains from dogs.

### DNA and amino acid sequence analyses

**Figure 2:**
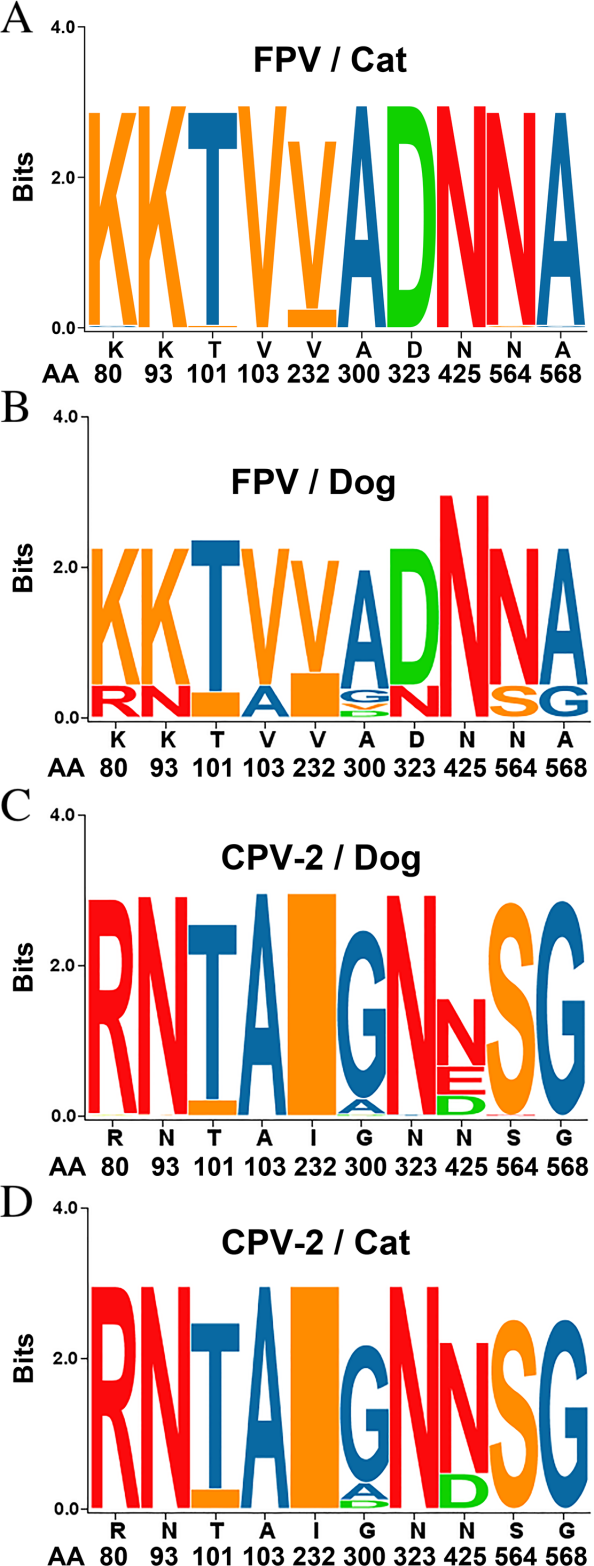
Sequence logos showing the amino acid usage in the VP2 of Carnivore *protoparvoviruses*. Sequence logos showing the amino acid usage in the VP2 of Carnivore *protoparvoviruses* (*n* = 600). (A) FPV in cat, (B) FPV in dog, (C) CPV-2 in dog and (D) CPV-2 in cat. The relative sizes of the letters in each stack represent their relative frequencies at each amino acid position. The height of the entire stack of residues is the information measured in bits (*Y*-axis): from minimum (0) to maximum conservation (3).

The entire VP2 nucleotide sequences were analyzed using DNASTAR software, revealing 96.8–98.5%, 99.8–100% and 99.3–100% homology with CPV-2 (2a, 2b and 2c) between our isolates and reference FPV strains, respectively. Amino acid sequence comparisons among the 6 isolates and the 23 reference strains revealed that the 6 isolates are similar to FPV based on the critical VP2 amino acid residues Lys80, Lys93, Val103, Asp232, Asn564 and Ala568. The critical residues for host range are also identical to those in FPV (Lys93, Val103, Ala300, Asp323) (Fig. S1). Amino acid variation in FPV VP2 sequences mainly occurs in the receptor-binding region (Fig. S1). Surprisingly, Thr101 was found in all of our FPV-like isolates and some of the recent reference strains, but the prototype FPVs carry Ile101 in their VP2 protein (Fig. S1). Sequences conservation was studied by applying a sliding window analysis for the entire VP2 sequences. Sequence logo analysis of the amino acid sequence of FPV in dogs were similar to FPV in cats (Fig. 2). These results confirm that the six samples isolated from dog feces in Vietnam in the present study are FPV-like viruses.

### Ile101Thr mutation changes the binding surface of the VP2 protein

**Figure 3:**
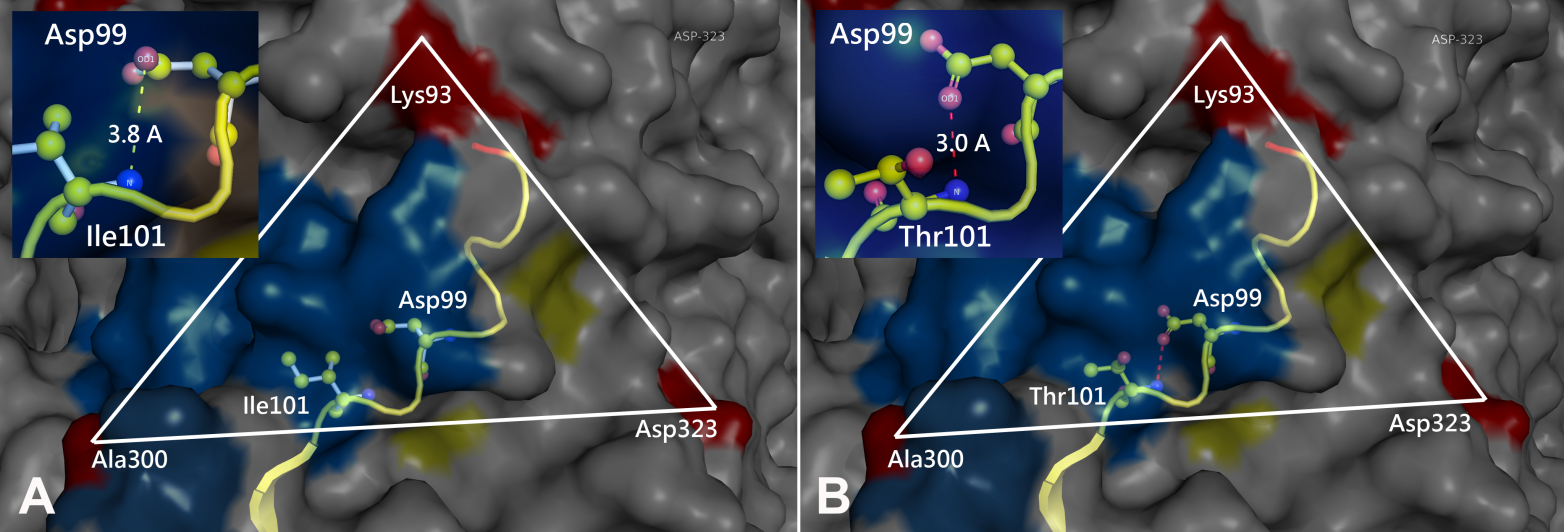
Comparison of the 3D structure of the transferrin receptor-binding site of the prototype FPV (A) and our isolate (B) capsid protein VP2. Comparison of the 3D structure of the transferrin receptor-binding site of the prototype FPV (A) and our isolate (B) capsid protein VP2. Red residues represent the transferrin receptor-binding site. Triangle areas indicate the VP2 footprint for the transferrin receptor. Blue residues represent capsid protein binding to Fab fragments of neutralizing antibodies. Schematic diagram of amino acids interactions are shown in the upper left corner.

**Figure 4:**
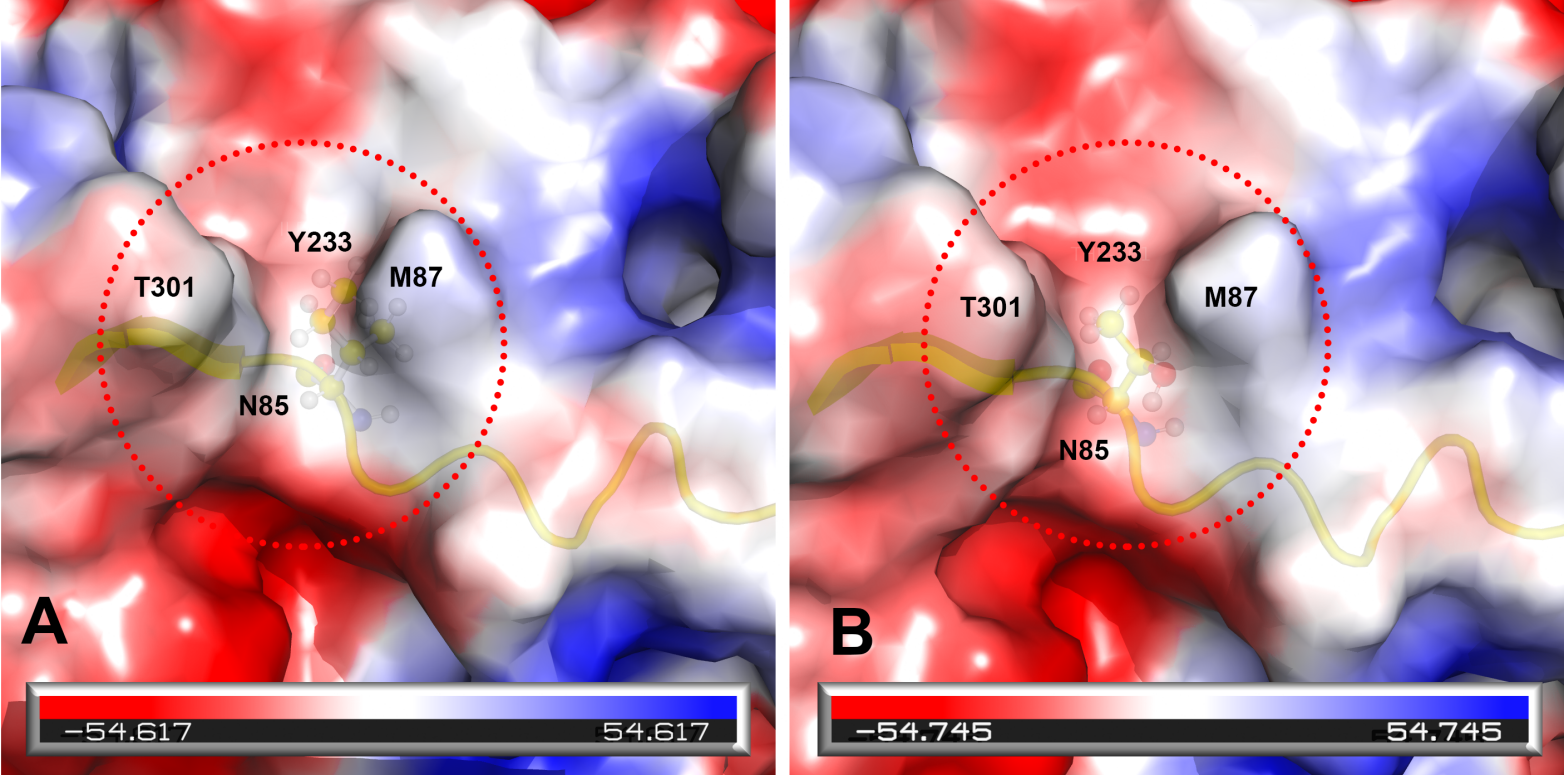
Analysis of electrostatic interactions on the molecular surface near residue Ile101 (A) compared to Thr101 (B) of the VP2 protein. Analysis of electrostatic interactions on the molecular surface near residue Ile101 (A) compared to Thr101 (B) of the VP2 protein. Red and blue represent negative and positive charge areas, respectively. Residues 93 to 104 of the VP2 protein are highlighted in yellow. Residue 101 is shown in ball & stick presentation.

Prototype FPV and our isolate were compared by protein structure modeling with SWISS-MODEL to analyze the transferrin receptor-binding surface. The 3D structure of the VP2 protein was constructed to identify residue 101 within the receptor-binding region (Fig. 3). Our results showed that residue 101 is located not only at the core of the receptor-binding region but also at the antibody-binding site (Fig. 3). Interestingly, the Ile101 to Thr101 mutation leads to the formation of a polar contact between Asp99 and Thr101, resulting in a change to the molecular surface of the core of the receptor-binding region (Fig. 3). Our results demonstrated that Thr101 contacts more negative charges than does Ile101 within this region (Fig. 4). The molecular surface near residue 101 forms a canyon structure surrounded by amino acids Asn85, Met87, Tyr233 and Thr301. The deep-buried mutated residue (101Ile → Thr) affects the charge distribution in this canyon area (dotted circular scale), which is located at the edge of the receptor binding site (Fig. 4). As this mutation may affect binding ability to the virus host cell receptor, Ile101Thr appears to alter the local protein contact potential of the VP2 protein.

## Discussion

FPV has been known as a contagious disease of cats since 1920s (Verge & Cristoforoni, 1928). CPV-2, considered a prominent host variant of the feline virus (Truyen, Platzer & Parrish, 1996), started to appear in dogs in the late 1970s. Although FPV has been revealed to be genetically stable (Decaro et al., 2008), CPV-2 demonstrates higher rates of nucleotide changes (Decaro et al., 2009; Pereira, Leal & Durigon, 2007; Shackelton et al., 2005). A few years after its discovery, the original CPV-2 was replaced by two antigenic variants, CPV-2a and CPV-2b, which can be differentiated by 5-6 amino acids of the main capsid protein VP2 (Parrish, 1991; Parrish et al., 1985). CPV-2c, the latest variant, was detected in 2000 in Italy (Buonavoglia et al., 2001), and its rapid spread to other continents has attracted increasing attention (Calderon et al., 2011; Chiang et al., 2016; Hong et al., 2007; Martella et al., 2004; Nakamura et al., 2004; Vannamahaxay et al., 2017; Wang et al., 2016; Yi et al., 2014).

To date, the transmission of CPV-2 to cats and FPV to dogs has been an interesting topic for researchers, and many in vivo and in vitro experiments have been conducted to provide more detail regarding the host ranges of these viruses. Based on abundant in vitro results, FPV efficiently replicates only in feline cells, whereas CPV-2 can replicate in both canine and feline cells (Hueffer et al., 2003b; Truyen & Parrish, 1992). Under natural conditions, CPV-2a, 2b and 2c have all been isolated from cats, with feline panleukopenia reported in many countries (Battilani et al., 2006; Decaro et al., 2010; Decaro et al., 2011; Ikeda et al., 2000; Mochizuki, Harasawa & Nakatani, 1993; Nakamura et al., 2001; Truyen, Platzer & Parrish, 1996). In contrast, information about FPV in dogs in the field is lacking. The host ranges of FPV and CPV-2 are quite sophisticated in vivo. Specifically, FPV can replicate in feline tissues, including the thymus, spleen, lymph nodes, and intestinal epithelial cells, and high viral loads are shed in feces. In dogs, the virus is found only in the thymus and bone marrow but is not detected in the mesenteric lymph nodes or gut, with no virus shedding in feces (Truyen & Parrish, 1992). Regarding viral evolution, the CPV-2 ancestor can only infect the gut, meaning it can be shed and spread widely among dogs (Truyen & Parrish, 1992).

As mentioned above, there is limited information to date on FPV infection in dogs in the field. In 1993, an isolate was recovered from a typical clinical parvoviral infection in a dog, but the virus possessed properties more similar to FPV than to CPV-2 or CPV-2a. The results suggest the possibility that transmission of FPV to dogs may have occurred (Mochizuki, Harasawa & Nakatani, 1993). Recently, transmission of FPV from cats to dogs in clinics was detected in Pakistan in 2018 (Ahmed et al., 2018) and in Thailand in 2019 (Charoenkul et al., 2019). Just a few FPV cases found in dogs during the past two decades. This means FPV has only occasionally infected dogs. In addition, mutation of apical domain residues in host TfR was observed to be critical for controlling parvovirus binding (Goodman et al., 2010; Hueffer et al., 2003a; Kaelber et al., 2012; Palermo, Hueffer & Parrish, 2003). The sequence of TfR from FPV-infected dogs needs to be further elucidated. Interestingly, according to phylogenetic analysis, the six specimens in our study cluster with FPV (Fig. 1), and all six isolates were collected from feces of dogs suspected of having canine parvoviral infection (Table 1). Comparison of the sequences of the six samples with the prototype FPV (M38246) and previously identified FPVs from Italy (EU498686) and China (DQ474237) showed high similarity in FPV VP2 among Vietnamese samples compared to other samples from around the world, with only some minor changes in the nucleic acid sequences. The important amino acid residues (80, 93, 103, 232, 323, 564, 568) and their corresponding nucleotide positions (nts 238-240, 277-279, 307-309, 694-696, 1690-1692, 1702-1704) can distinguish between FPV from CPV-2, with results similar to the careful examination by Decaro (Decaro & Buonavoglia, 2012). Taken together, our results are consistent with previous studies, indicating that FPV-like viruses can infect dogs in the field.

Due to the antigenic and genetic similarity between CPV-2 and FPV (Appel, Scott & Carmichael, 1979), both viruses can be classified as host-range variants of FPV (Ikeda et al., 2000). Moreover, other reports emphasize that the canine and feline host ranges can be determined by VP2 protein residues 93, 103, 300 and 323 (Truyen et al., 1995). Amino acid variations of FPV VP2 mainly occur in the receptor-binding region (Fig. S1), indicating that those variations may be involved with the interaction between the virus and its receptor. Residues Met87Leu, Ile101Thr, Ala300Gly, Asp305Tyr and Val555Ile of the VP2 protein have been identified as important for host range (Miranda & Thompson 2016). Surprisingly, our results showed that a Thr101 mutation in all of our FPV-like isolates but not in prototype FPV strains, as prototype FPV and CPV-2 encode Ile101 (Miranda & Thompson, 2016). According to the concept of consecutive genetic evolution of CPV-2, the Ile101 to Thr101 mutation was found in all CPV-2 variants (CPV-2a, 2b and 2c). Interestingly, the Thr101 mutation was also observed in recent FPV isolates in nature. Therefore, this particular mutation, Thr101, has been reported in circulating FPV and CPV-2 populations (Hoelzer et al., 2008). Whether the Thr101 variant is able to replicate efficiently in the gastrointestinal tract of dogs needs to be further investigated. Overall, there is limited information about the 3D structure or binding surface for Thr101 in the VP2 protein. Our 3D structure results indicated that the Thr101 mutation leads to the formation of a polar contact between Asp99 and Thr101. This may result in a change to the molecular surface of the core of the receptor-binding region, subsequently influencing receptor-binding affinity between the transferrin receptor and the virus. In addition, our data agree with previous studies indicating that residue 101 is located not only at the core of the receptor-binding region (Lee et al., 2019) but also at the antibody-binding site (Organtini et al., 2016). Taken together, the impact of the Thr101 mutation will require further investigation.

## Conclusions

This study indicates the appearance of FPV-like strains in Vietnamese dogs. These FPV-like isolates carry an Ile101Thr mutation that is different from prototype FPV strains. Our data provide evidence for the existence of changes in the charge, protein contact potential and molecular surface of the core of the receptor-binding size with an Ile101 to Thr101 mutation. This is also the first study to provide reliable evidence that the FPV may be a threat to the Vietnamese dog population, which emphasizes the necessity of raising awareness for surveillance of this virus.

##  Supplemental Information

10.7717/peerj.9752/supp-1 Supplemental Information 1 Multiple amino acid sequence alignment of VP2 (a.a. position 70-140 and 280-350) of FPVs and CPV-2 variants Stars represent residues that can be used to distinguish between FPVs and CPV-2 variants. The red color represents residues that can determine the canine or feline host range. Bold font represents residue Ile101 or Thr101. FPV-like isolates in the present study are indicated as a solid line box. The numbering at the top of the alignment is based on the FPV VP2 sequence.

## Additional Information and Declarations

### Data Availability

The following information was supplied regarding data availability: Our raw data are the viral sequences, which submitted to GenBank accession number MK357738 to MK357743.
