# Supplementary figures and images for: Genetic characterization of feline panleukopenia virus from dogs in Vietnam reveals a unique Thr101 mutation in VP2
Source: PeerJ. 2020 Oct 12;8:e9752. doi: 10.7717/peerj.9752 (PMC7560322; doi:10.7717/peerj.9752)

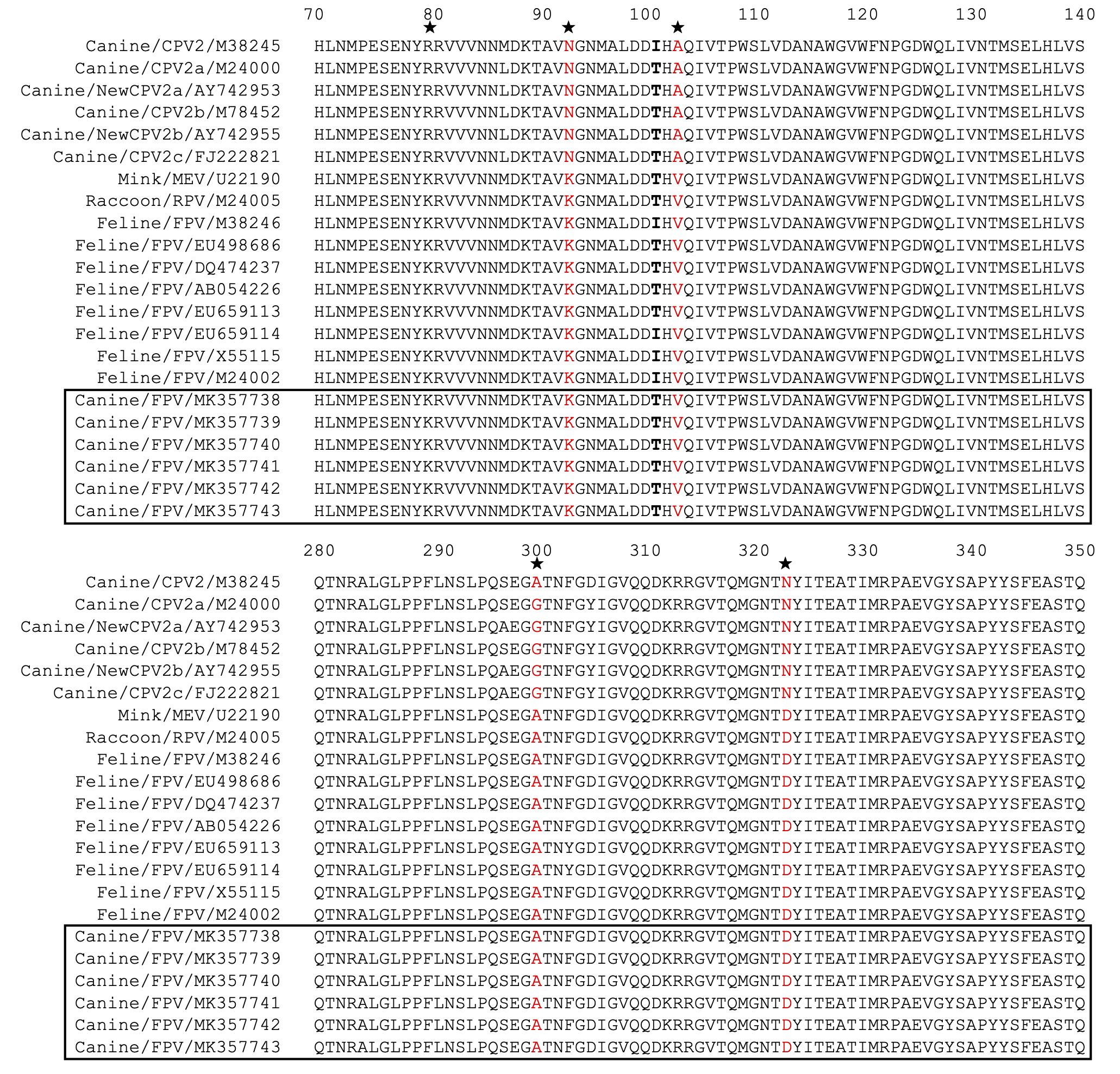

Supplement: Supplemental Information 1 — Stars represent residues that can be used to distinguish between FPVs and CPV-2 variants. The red color represents residues that can determine the canine or feline host range. Bold font represents residue Ile101 or Thr101. FPV-like isolates in the present study are indicated as a solid line box. The numbering at the top of the alignment is based on the FPV VP2 sequence. [file peerj-08-9752-s001.jpg]
